# Adaptive Machine Learning Approach for Importance Evaluation of Multimodal Breast Cancer Radiomic Features

**DOI:** 10.1007/s10278-024-01064-3

**Published:** 2024-03-13

**Authors:** Giulio Del Corso, Danila Germanese, Claudia Caudai, Giada Anastasi, Paolo Belli, Alessia Formica, Alberto Nicolucci, Simone Palma, Maria Antonietta Pascali, Stefania Pieroni, Charlotte Trombadori, Sara Colantonio, Michela Franchini, Sabrina Molinaro

**Affiliations:** 1 Institute of Information Science and Technologies “A. Faedo” (ISTI), National Research Council of Italy (CNR), Pisa, Italy; 2 Institute of Clinical Physiology (IFC), National Research Council of Italy (CNR), Pisa, Italy; 3 Department of Computer Science, University of Pisa, Pisa, Italy; 4 Policlinico Gemelli IRCCS, Rome, Italy; 5 Università Cattolica del Sacro Cuore, Rome, Italy; 6 Studi Michelangelo srl, Firenze, Italy

**Keywords:** Breast cancer, Radiomic, Adaptive feature selection, Model reduction

## Abstract

Breast cancer holds the highest diagnosis rate among female tumors and is the leading cause of death among women. Quantitative analysis of radiological images shows the potential to address several medical challenges, including the early detection and classification of breast tumors. In the P.I.N.K study, 66 women were enrolled. Their paired Automated Breast Volume Scanner (ABVS) and Digital Breast Tomosynthesis (DBT) images, annotated with cancerous lesions, populated the first ABVS+DBT dataset. This enabled not only a radiomic analysis for the malignant vs. benign breast cancer classification, but also the comparison of the two modalities. For this purpose, the models were trained using a leave-one-out nested cross-validation strategy combined with a proper threshold selection approach. This approach provides statistically significant results even with medium-sized data sets. Additionally it provides distributional variables of importance, thus identifying the most informative radiomic features. The analysis proved the predictive capacity of radiomic models even using a reduced number of features. Indeed, from tomography we achieved AUC-ROC $89.9\%$ using 19 features and $92.1\%$ using 7 of them; while from ABVS we attained an AUC-ROC of $72.3\%$ using 22 features and $85.8\%$ using only 3 features. Although the predictive power of DBT outperforms ABVS, when comparing the predictions at the patient level, only 8.7% of lesions are misclassified by both methods, suggesting a partial complementarity. Notably, promising results (AUC-ROC ABVS-DBT $71.8\%$-$74.1\%$) were achieved using non-geometric features, thus opening the way to the integration of virtual biopsy in medical routine.

## Introduction

Breast cancer (BC) is the leading cause of death among women, and according to the Global Burden of Disease 2019, one in every eight new cancer cases was diagnosed as BC, making it the world’s top most prevalent type of cancer [1]. There are 5 stages of BC, ranging from the non-invasive ductal carcinoma in situ (stage 0) to the more invasive ones (stages I–IV). To date, stages 0 and I exhibit an almost 100% 5-year survival rate, in contrast to stages II and III which show survival rates of 93% and 72% respectively [1]. Hence, the detection and classification of early-stage BC has a crucial impact on patients’ prognosis, as it may allow for less invasive surgical procedures.

The screening procedure relies on the assessment of radiological images, essentially based on mammography (MX) [2, 3], breast ultrasound (US) [4, 5], or contrast-enhanced magnetic resonance imaging (DCE-MRI) [6]. Nowadays, the cornerstone technique for BC screening is MX, which can reduce the mortality rate by 20–22% [7]. However, integrating MX with additional techniques (e.g., Digital Breast Tomosynthesis (DBT), US, or Automated Breast Volume Scanner (ABVS)) can significantly improve detection capability [8, 9]. In particular, DBT by eliminating the problem of tissue overlap and allowing enhancing the identification of parenchymal distortions, increases the Cancer Detection Rate (CDR) of breast lesions by 2.7/1000 compared to MX alone (CDR 5.3/1000) [10]. Also, US-based imaging techniques can improve CDR (4.9 per 100 in a population of women with MX-dense breast) at the cost of a higher false positive rate than DBT [11]. Among the US-based radiological methods, ABVS is a screening technique (for patients with intermediate risk and with MX dense breasts) characterized by a greater reproducibility compared to traditional US [12–14].

While imaging methods are primarily used for screening, biopsy is the only existing tool to classify a breast lesion as benign or malignant and to characterize the malignant ones by receptor expression/phenotype (ER, PR, and HER2 receptor). However, the biopsy is an invasive, time-consuming, and expensive procedure that can cause anxiety and discomfort to the patient and it’s also frequently done needlessly, even for lesions that could be benign [15]. To overcome the cost and limitations of biopsy, the ultimate goals of modern breast imaging encompass the early detection of BC, followed by the accurate classification of the lesion and the prediction of its clinical course and biological aggressiveness.

Among modern image-based mathematical approaches, radiomics is a quantitative approach which uses automated methods to extract valuable information from radiological images. By selecting the most relevant features and embedding them in a Machine Learning (ML) pipeline, radiomics enables the development of predictive models that support standard radiological techniques [16, 17] (e.g., to assess the aggressiveness of cancer lesions). Several published studies have highlighted the potential of radiomics in addressing medical challenges in BC care, such as early detection, classification, cancer sub-type determination and molecular profiling, prediction of lymph node metastases, and prognostication of treatment response [2–6, 18–21].

Despite the large number of published studies, most of the proposed strategies extract quantitative parameters from databases of unimodal medical images (such as DCE-MRI [6, 22], MX [2, 19], and ABVS [14, 23]). Only a minority obtains acquisition from multiple techniques, including DWI + DCE-MRI [18], ABVS + Elastography [24], and BM-US + Elastography [25]. However, there is a paucity of radiomic studies in the scientific literature using ABVS images. Although ABVS and DBT diagnostic performance have been previously compared [26], to the best of our knowledge there is no multimodal ABVS + DBT comparative radiomic analysis.

Difficulties in obtaining paired data, especially with modern techniques like ABVS, lead to undersized study databases. This difficulty exacerbates the already known problems of radiomic studies, in particular, the risk of overfitting given the high ratio of radiomic features to sample size [27–30], and requires ad hoc techniques to maximize information extracted from the database without data leakage.

This work presents a methodological approach for studying radiomic databases of moderate sample size. The approach involves pre-selecting features through stability analysis, designing a validation scheme to maximize extracted information (using nested leave-one-out, LOO, cross-validation), and generating distributed importance scores to define an adaptive augmentation procedure. We aimed to differentiate malignant from benign mass lesions using the radiomic features extracted from a medium-sized multi-modal dataset, including DBT and ABVS breast images [31]. To the best of our knowledge, the P.I.N.K database is the first to include both ABVS and DBT acquisitions for each patient. The data, collected in an ongoing longitudinal multicentre study, allow us for a rigorous and significant comparative study of the predictive capabilities of ML models trained on the different modalities.

The paper is structured as follows. The “Materials and Methods” section discusses the characteristics of the population and the collection protocol. The analysis of trait stability and the consequent reduction of independent features is included. We then present the nested LOO method, adapted to this database, for generating the distributional feature importance, exploited to select a minimal model using an adaptive procedure. The "Results" section show the features most stable to perturbations and the scores of the trained models: the one with all (non-collinear) features, the model obtained by an adaptive procedure, and the one trained on texture features only. The proposed approach and the related results enabled to draw medical conclusions. Finally, future work may involve the idea of a virtual biopsy, which integrates the texture features-based information with the patient’s medical history.

## Materials and Methods

### Study Population and Acquisition Protocol

The database used in this work, as defined in the P.I.N.K study protocol, includes 66 women over 40 years of age who have both DBT and ABVS acquisition in concurrent periods. The women presented spontaneously for routine breast examination at 2 diagnostic centers in Italy, both equipped with DBT (vendor-independent tomosynthesis — Siemens, GE, Hologic) and ABVS (ABVS ACUSON S2000TM — Siemens Medical Solutions, Inc, Mountain View, CA) devices. The DBT data collect cranio-caudal scans, while the ABVS images are mainly anterior-posterior views. Exclusion criteria include the presence of breast implants, pregnancy, or breastfeeding.

**Table 1:** Women’s distribution by age and breast density. Scores reported as malignant/(malignant + benign)

**Age classes**	**Breast density (Bi-RADS levels)**				Total
	*A*	*B*	*C*	*D*	
*< 50 ys*	-	3/4	8/15	0/6	11/25
*50–59 ys*	1/1	2/4	4/13	2/3	9/21
*60–69 ys*	2/2	3/5	2/3	-	7/10
*> 70 ys*	1/2	4/4	3/4	-	8/10
Total	4/5	12/17	17/35	2/9	35/66

The ground truth used to train the model is binary tumor classification (malignant/benign, see Table 1), which is obtained from post-intervention histological data (62.3%), if available, or pre-intervention histological data (37.7%), otherwise. The (66) patients included in our study all have mass-forming lesions ($n = 69$), visible both in the DBT images and in the ABVS images.

### Lesion Segmentation

Lesion segmentation in ABVS and DBT images was performed by three radiologists (with over 5 years of experience) in consensus using 3D Slicer [32], a free open-source software platform for image analysis and visualization. To ensure a rigorous segmentation process, breast radiologists followed a strict pipeline, consisting of the following: (i) identifying the lesion in each ABVS and DBT image, (ii) manually delineating the contour of the lesion using annotation tools such as brush and intensity threshold, (iii) assessing the quality of each 2D mask, considering also the lesion volume and shape, (iv) refining and cleaning each segmentation (e.g., holes with an area < 9 pixels have been filled, and regions of less than 30 pixels have been removed; when necessary, the borders have been smoothed by applying to the segmentation mask a binary closing as implemented in the Multidimensional image processing^1^), (v) validating the lesion segmentation.

**Fig. 1:**
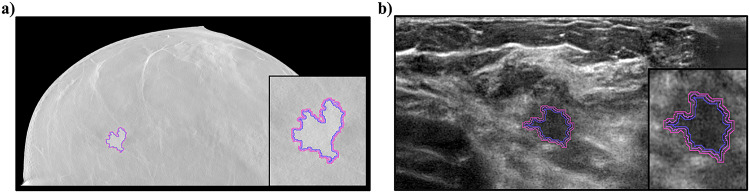
The same lesion segmented for both modalities: **a** DBT and **b** ABVS. The manual segmentation (the standard one) has been slightly modified by applying standard morphological operators to produce two different annotation masks (i.e., reduced and increased) and assess the feature robustness against small variations, as reported in the “Features Extraction, Selection, and Stability Analysis” section

### Features Extraction, Selection, and Stability Analysis

Twenty-five radiomic features, both geometric (e.g., Volume, Sphericity) and textural (e.g., Total energy, Coarseness) were extracted using Pyradiomics v.3.0.1 [33]. These features were computed exclusively from the *original images* and are not analytically correlated (Fig. 3). We decided to work with a subset of radiomics features computed on original images in order to follow the guidelines of the Image Biomarker Standardization Initiative (IBSI, https://theibsi.github.io/ibsi2/), which have not been released yet for filtered images [34].

The 25 features were subjected to a Principal Component Analysis (PCA) to assess the cardinality of the principal components and thus to identify the presence of linearly correlated features. To determine a subset of features that are non-redundant and stable to small variations, we used the following procedure: (i) the segmentation annotated by radiologists (i.e., *standard*) has been used as the reference mask, (ii) each radiomic feature has been tested varying both the bin width^2^ It is defined as follows: $\left( max (\text {gray level}) - min(\text {gray level})\right) / \#bins$. and the mask type (reduced, standard, increased), as shown in Fig. 1, (iii) for each combination of bin width (*bw* $\in$ {15,20,25,30,35}) and mask (*m* $\in$ {reduced, standard, increased}), the instability of the $i^{\text {th}}$ feature $\Delta _i$ is estimated as follow:

1 $$\begin{aligned} \frac{1}{15\cdot \# p} \sum _{m, bw, p} \frac{|\text {f}_i\left( m, bw, p \right) -\text {f}_i\left( st, 25, p \right) |}{{\textbf {max}}_{p^*}\left( \text {f}_i\left( st, 25, p^* \right) \right) - {\textbf {min}}_{p^*}\left( \text {f}_i\left( st, 25, p^* \right) \right) } \end{aligned}$$

where $st:= standard$, $\#p$ the number of patients, and $f_i(m,bw,p)$ is the $i^\text {th}$ feature calculated using the mask size *m*, the bin width *bw* on the patient *p*, (iv) between two highly correlated features, the most unstable is defined as the one with the higher value of $\Delta$ and therefore is dropped. After variable reduction, PCA was repeated to ensure the redundancy of the dropped features.

### Nested LOO Cross-Validation

Tumor classification was performed using three ML approaches: Random Decision Forest (RDF, an ensemble of $n_t$ random trees, with $n_t \in [50,250]$), polynomial Support Vector Machine (SVM, with a polynomial kernel of degree 3 with a cost of $c \in [1,10]$), and Logistic Model (Logit, binary classifier based on a binomial general linear model). These methods were trained on both DBT- and ABVS-derived radiomic features. However, the high ratio ($\sim 1/3$) between the number of independent features and the sample size, as well as the high variability of the population under study (regarding lesion shape, breast density, extension, branching, etc.), require the use of a robust ad hoc technique for the optimization of the classifier’s hyperparameters (i.e., the number of trees for the RDF and the cost for the SVM) and the subsequent evaluation of the performance of the models.

**Fig. 2:**
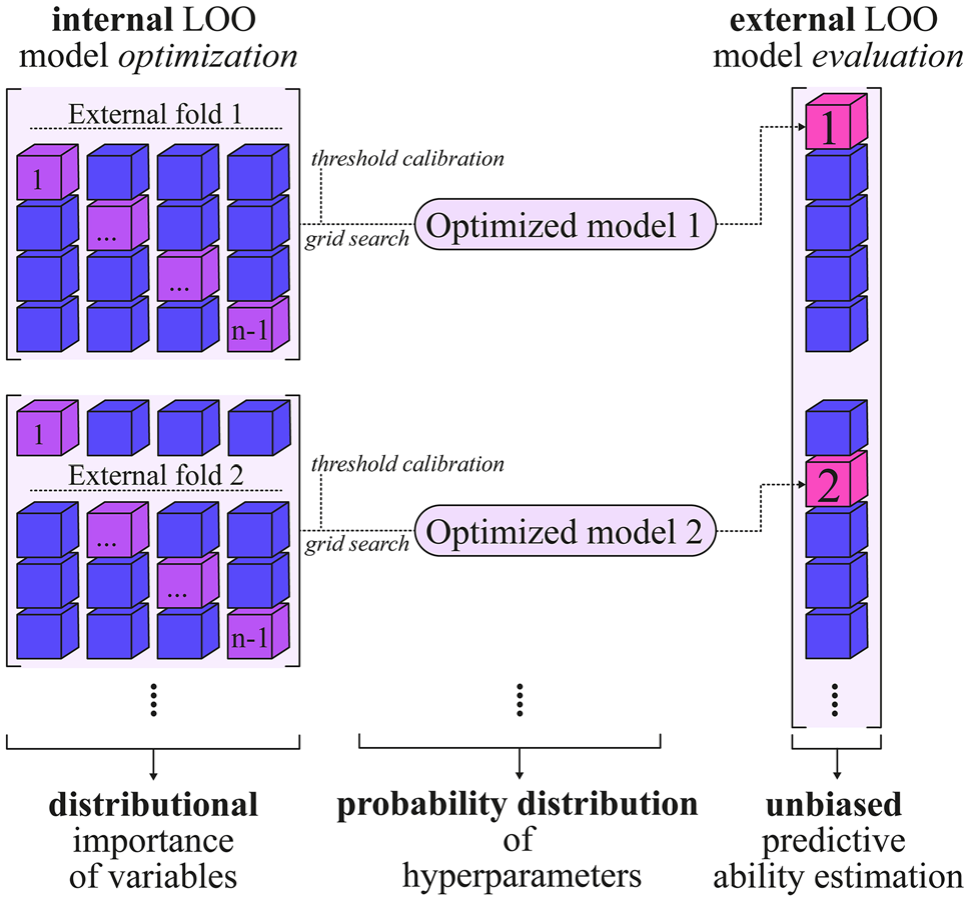
Optimization and validation scheme adapted to the dimension $(n=69)$ of the dataset. The external LOO model evaluation uses a leave-one-out approach to provide an estimate of performance for each patient, at the cost of increased computational complexity. Each model evaluation is performed after an optimization (i.e., internal LOO) using an additional LOO strategy combined with a grid search for the optimal hyperparameter. To reduce positively biased estimates, every optimized model calibrates its internal threshold

Referring to Fig. 2, a LOO nested cross-validation approach was defined as an extension of classical nested cross-validation [35]. In order to adapt the implementation to the available data, the described procedure was implemented in-house from scratch using R (v4.2.2) and Python (v3.9.13). The dataset (of cardinality *n*) is partitioned into *n* variants (called external LOOs) by a leave-one-out scheme. For each external LOO, $n-1$ data points are used as training. One data point is treated as an independent evaluation to estimate the generalization ability of the model. The model used in each step of the external LOO (i.e., optimized model i) is obtained by calibrating the hyperparameters using a grid search. The latter is defined as the one that maximizes the performance (i.e., the AUC-ROC, Area Under the Receiver Operating Characteristic Curve) in the internal LOO cross-validation. The same internal LOO cross-validation is used to estimate the $i^\text {th}$ optimal classification threshold for the corresponding model (i.e., the probability value that discriminates categories) as the one maximizing the sum of specificity and sensitivity across the $n-1$ LOO evaluations. The $i^\text {th}$ external LOO model evaluation then performs a prediction estimate based on the optimized $i^\text {th}$ model (grid search + internal LOO cross-validation) with the appropriately calibrated $i^\text {th}$ threshold.

This approach provides a low-bias estimate of the model’s generalization capability from the external LOO procedure, where the data employed is never used in the optimization and training phases and is therefore not affected by data leakage. Similarly, to further reduce positively biased results, the optimal threshold is calibrated for each model (internal LOO model optimization). In addition, unlike the canonical partitioning of the available data into training, validation, and test set required for both optimization and external validation, the use of nested leave-one-out cross-validation makes the most of the information content of this dataset.

A major drawback is that the computational cost of nested LOO cross-validation is quadratic in dataset cardinality. Indeed, for each choice ($\#\text {GridSearch}$) of hyperparameters to be adjusted, each optimization of the internal LOO model requires $n-1$ model training sessions. For each external LOO model evaluation, this procedure must be performed *n* times. The total number of model training sessions is as follows: $\#\text {GridSearch}\cdot n\cdot (n-1)$.

### Distributional Feature Importance and Adaptive Selection

The optimization/validation scheme used does not produce a single optimal model, but rather a family of models (i.e., $n=69$ different models generated by the external LOO, one for each element in the dataset). Each of these models is obtained by nested LOO cross-validation and is the optimized model on the remaining $n-1$ (68) training data. The relative importance of the features can be calculated in an analytical way for each trained model. Features importance are as follows: (i) the Mean Decrease Gini/Accuracy for RDF, (ii) SVM coefficient multiplied by its support vector for SVM, (iii) change in deviance (i.e., reduction of prediction error) for Logit. The external LOO scheme can be used to compute the probability distribution of the importance for each input feature (obtained from the $n=69$ LOO-models) which is more informative than a canonical point estimate obtained from a single retrained model. Furthermore, the resulting distributions can be used to analyze the stability of the model. Indeed, low distribution variance corresponds to a feature whose relative importance is constant across trained models.

The resulting relative importance is used to define an adaptive procedure for the selection of a subset of the features.

**Algorithm 1:**
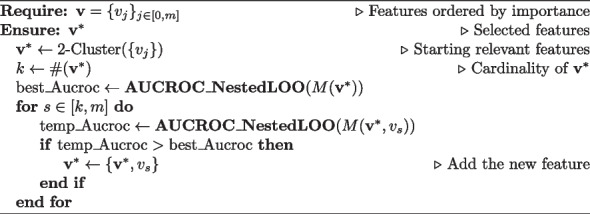
Adaptive feature selection

A 2-clustering algorithm divides the features into 2 groups (most relevant/less relevant), as reported in Algorithm 1. The most relevant features are added to the model, whose performance is evaluated using the nested LOO cross-validation procedure previously introduced. The performance score used is the LOO-AUCROC obtained from the external LOO cycle (summarized by the function **AUCROC_NestedLOO** in the algorithm). The procedure adds the features to the model in the order of their relative importance (mean decrease accuracy). After adding each feature, the performance score is evaluated: if the model performs better, the feature is retained; if not, it is dropped. This procedure returns the minimal model with the best performance by performing a total of $86940*19 \sim 2\cdot 10^6$ simulations ($\sim 13$ h on 1.6 GHz Dual Core Intel i5 CPU). In contrast, a brute-force exploration of all the combinations of features would have required $86940\cdot 2^{19} \sim 10^{10}$ simulations.

## Results

### Feature Selection, Redundancy Correction, and Stability Analysis

**Fig. 3:**
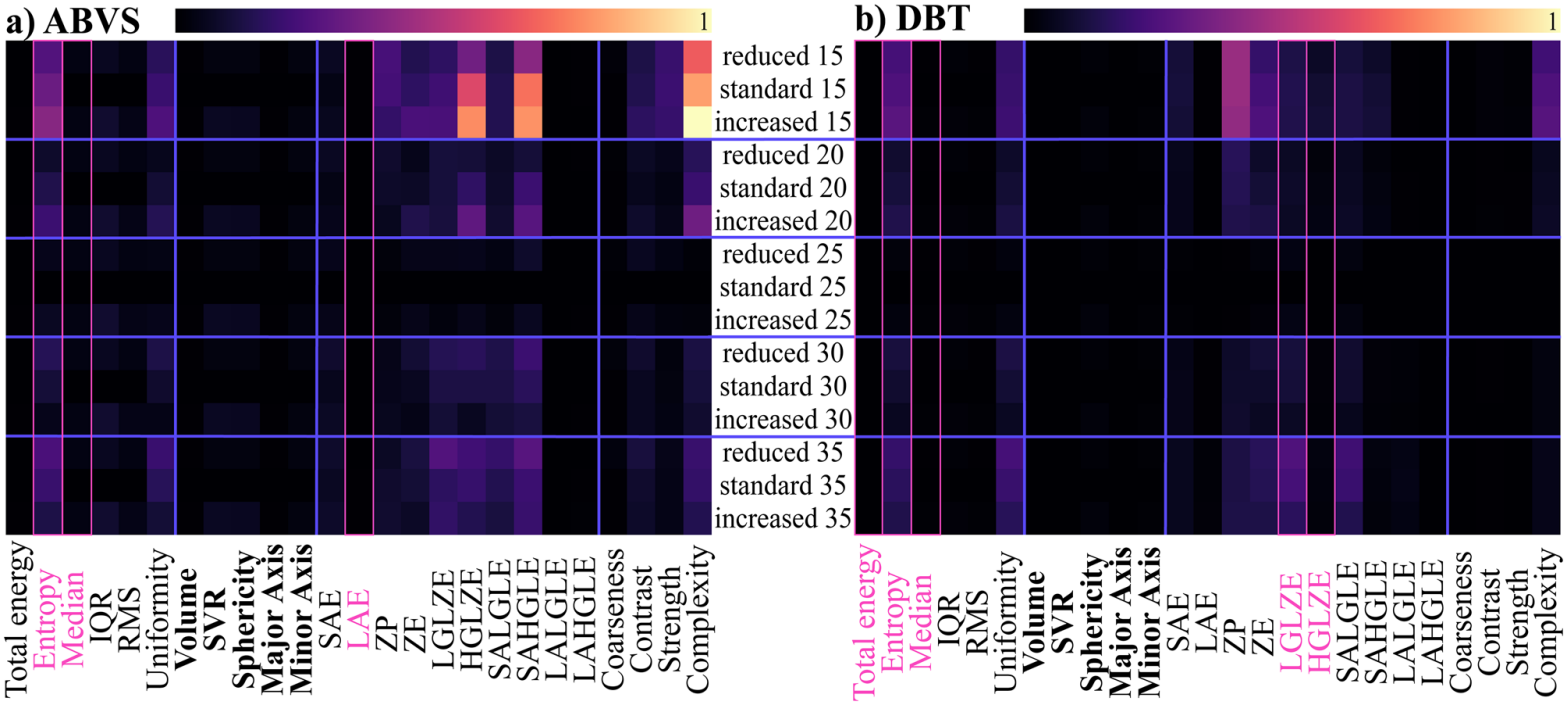
The score defined in Eq. 1 has been computed to assess the radiomic feature stability for ABVS (panel **a**) and DBT (panel **b**). Each row corresponds to a different extraction: reduced/standard/increased represents which mask was used in the computation, while the numbers [15–35] are bin width used to extract the features. Geometric features are shown in bold. Features dropped after the redundancy correction are marked with a pink box

Among the starting subset of original features reported in Fig. 3, we performed a selection based on the elimination of redundant ones. In particular, within pairs of correlated variables (Pearson Correlation above 0.95), we decided to keep the feature more stable with respect to the stability measure (“Features Extraction, Selection, and Stability Analysis” section).

The PCA analysis showed that the number of principal components (99% of the cumulative variance) does not change before and after the redundancy correction (16 components for ABVS and 14 for DBT).

For ABVS, redundant features are as follows: the Intensity Histogram Entropy (highly correlated with the Intensity Histogram Uniformity), the Median (highly correlated with the Root Mean Squared (RMS)) and GLSZM LAE — Large Area Emphasis (highly correlated with GLSZM LALGLE — Large Area Low Gray Level Emphasis). For DBT, redundant features are as follows: the Total Energy (highly correlated with the Volume), the Intensity Histogram Entropy (highly correlated with the Intensity Histogram Uniformity), the Median (highly correlated with the Root Mean Squared (RMS)), GLSZM LGLZE — Low Gray Level Zone Emphasis (highly correlated with GLSZM SALGLE — Small Area Low Gray Level Emphasis), GLSZM HGLZE — High Gray Level Zone Emphasis (highly correlated with GLSZM SAHGLE (Small Area High Gray Level Emphasis).

Figure 3 represents the heat maps of the stability of the features with respect to the bin width and the perturbation (increase/decrease) of the segmentation mask. They show that the shape features (in bold) are generally more stable compared to the texture ones. Notably, the least variability induced by geometrical perturbation of the mask is obtained with the default value of Pyradiomics for the bin width (25).

### Full Models: ABVS-DBT Comparison

The performance of the models trained on the same set of patients (called respectively **RDF-ABVS/SVM-ABVS/Logit-ABVS Model** and **RDF-DBT/SVM-DBT/Logit-DBT Model**) are reported in Table 2.

For the three models, the DBT Models always outperform the ABVS ones. Indeed, DBT-based models have a higher AUC-ROC compared to ABVS for each of the trained ML methods (69.9/73.0/89.9% vs 67.8/66.7/72.3% for SVM/Logit/RDF respectively). DBT is also better than ABVS at identifying pathological cases for all three models (94.7/81.6/84.2% vs 71.0/55.3/68.4% for SVM/Logit/RDF respectively), while the number of false positives is comparable between DBT and ABVS. The comparison between the three ML models highlights the strength of ensemble methods, with RDF proving to be the most accurate in almost all performance metrics, particularly for DBT data (AUC-ROC $89.9\%$, Accuracy/Specificity/Precision and Recall $>80$%). In addition, the simplest model (Logit) has very low accuracy, precision and recall.

**Table 2:** Full RDF-, SVM-, and Logit-ABVS and DBT Models. The best full model (RDF) is also retrained on the texture feature only (RDF-ABVS tx. and RDF-DBT tx.) and using the adaptive feature selection strategy. The performance metrics are computed by selecting the threshold using the inner nested LOO evaluation

	**AUC-ROC**	**Accuracy**	**Specificity**	**Precision**	**Recall**
**SVM-ABVS**	67.8%	68.1%	64.5%	71.0%	71.0%
**SVM-DBT**	69.9%	72.5%	58.0%	67.9%	**94**.**7**%
**Logit-ABVS**	66.7%	58.0%	61.3%	63.6%	55.3%
**Logit-DBT**	73.0%	73.9%	64.5%	73.8%	81.6%
**RDF-ABVS**	72.3%	68.1%	67.7%	72.2%	68.4%
**RDF-DBT**	**89**.**9**%	**80**.**7**%	**80**.**7**%	**84**.**2**%	84.2%
**RDF-ABVS tx.**	71.8%	71.1%	77.1%	70.3%	62.1%
**RDF-DBT tx.**	74.1%	74.4%	82.9%	75.0%	65.5%
**RDF-ABVS Reduced**	85.8%	76.8%	71.0%	77.5%	81.6%
**RDF-DBT Reduced**	**92**.**1**%	**85**.**5**%	**87**.**1**%	**88**.**9**%	84.2%

**Fig. 4:**
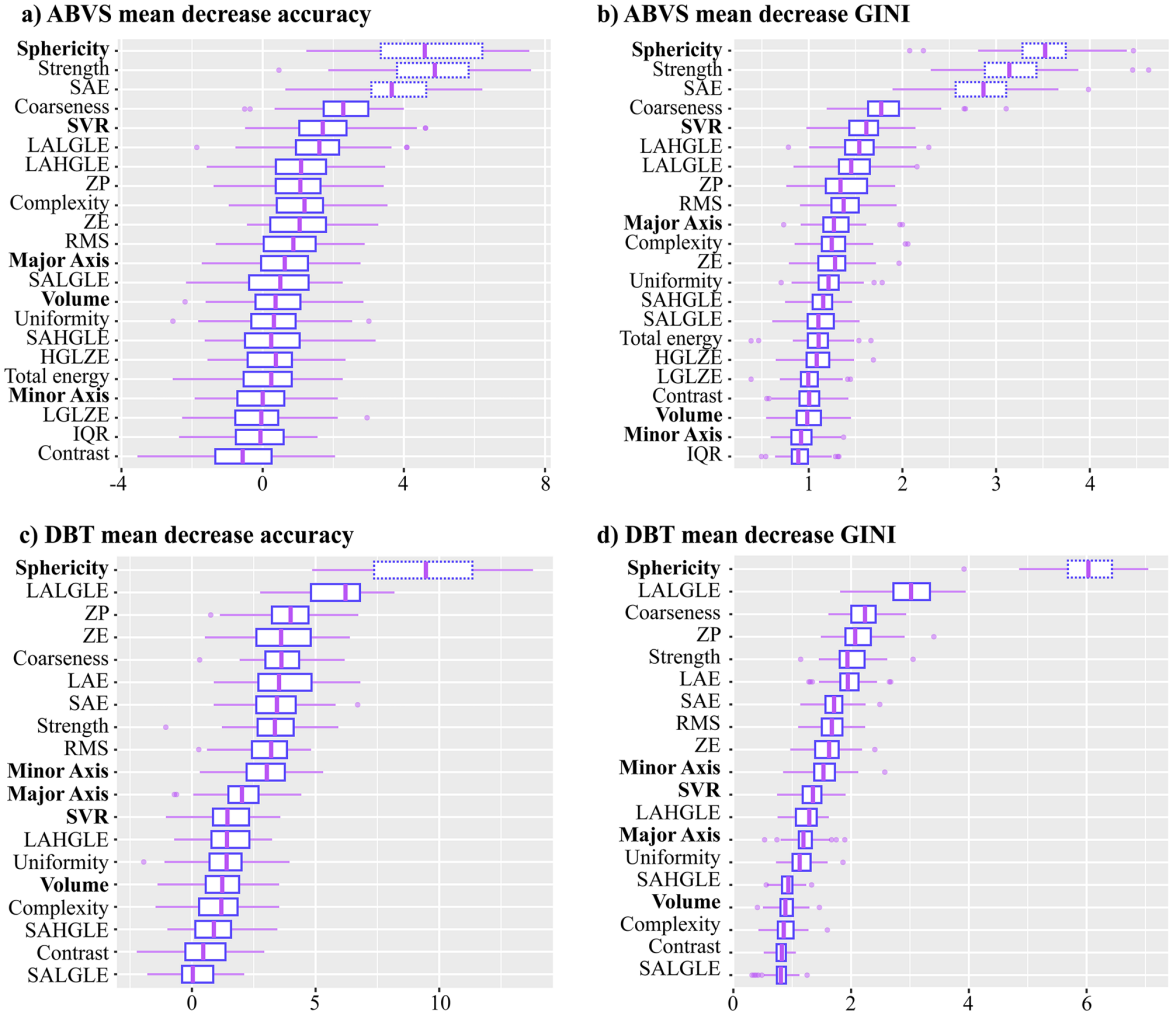
Distributional Feature Importance of RDF-ABVS analysis (panels **a**, **b**) and RDF-DBT (panels **c**, **d**). The scores are reported as mean value and IQR (3° and 4° quantiles), calculated from the nested LOO external procedure. Dashed boxes indicate features that are significantly more relevant features (Sphericity, SAE, and Strength for ABVS, while Sphericity for DBT)

As reported in the “Distributional Feature Importance and Adaptive Selection” section, we calculated the Distributional Feature Importance for DBT and ABVS (see Fig. 4). In terms of the most effective model, it emerges that Sphericity is the most important feature and, particularly for DBT acquisitions, geometric features play an important role in model classification. Among the other features, glszm SAE (Small Area Emphasis) and Strength, are relevant for Full ABVS Model. The high concordance of Mean Decrease Accuracy and Mean Decrease Gini is a good indicator of models stability. Similar results are obtained for the SVM, with sphericity being the most important feature for ABVS and DBT. Conversely, Logit mainly uses non-geometric NGTDM features to make its prediction, but this leads to unreliable predictions and affects both model accuracy and specificity.

The uniqueness of the P.I.N.K dataset (consisting of a dual DBT+ABVS acquisitions) allows for a comparison of the most effective models (RDF) at the patient level. It can be verified that 26.1% of the lesions (4 malignant cases and 8 benign cases correctly predicted *only by* DBT and 3 malignant cases and 3 benign cases correctly predicted *only by* ABVS) are correctly identified by only one of the two models. Conversely, only 8.7% (3 malignant and 3 benign cases) are misclassified by both models. Consequently, even considering the better performance of the Full RDF-DBT Model compared to the RDF-ABVS one, this suggests that the two modalities are partially complementary.

### Reduced Models: Adaptive Features Selection

An optimal set of the non-collinear features (Fig. 4) was identified by applying the Adaptive Selection Algorithm 1 to the most effective full model (i.e., ABVS/DBT RDF). The corresponding models trained on these subsets are the **RDF-ABVS Reduced Model** and the **RDF-DBT Reduced Model**.

**Fig. 5:**
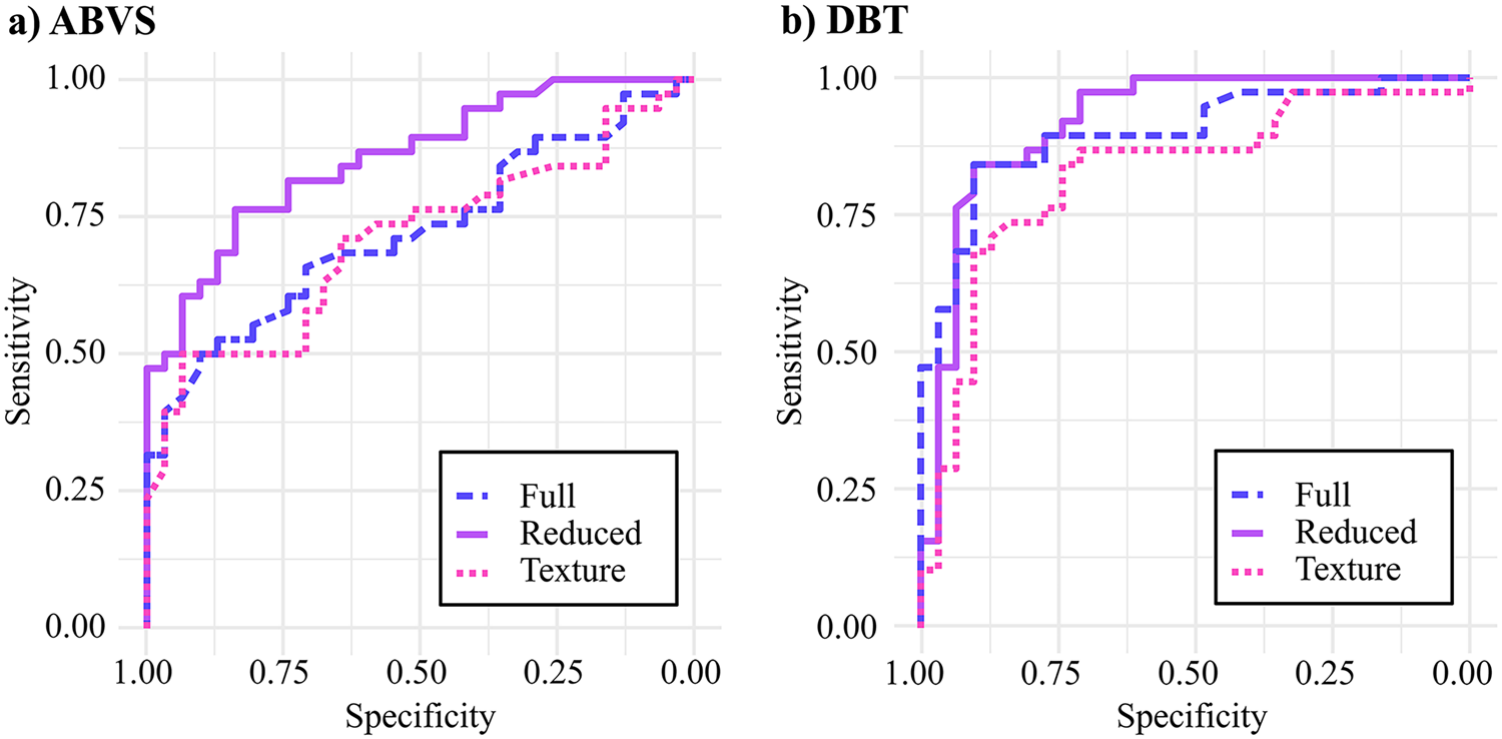
Receiver Operating Characteristic (ROC) curves for RDF-ABVS (panel **a**) and RDF-DBT (panel **b**). These curves represent the performance on the LOO external validation for the Full RDF model (trained on the whole set of radiomic features), the Reduced RDF model (features obtained from the adaptive procedure), and the RDF Texture one (without geometrical/shape features)

Starting from *Sphericity* for DBT and *Sphericity, glszm SAE, and Strength* for ABVS, the procedure iteratively adds only features with positive AUC-ROC contribution. The reduced ABVS model has an AUC-ROC of 85.8% using only the following 3 starting features: Sphericity, SAE (Small Area Emphasis), and Strength. On the other hand, the reduced DBT model uses 7 features to obtain an even higher AUC-ROC of 92.1%: Sphericity, LALGLE (Large Area Low Gray Level Emphasis), ZP (Zone Percentage), ZE (Zone Entropy), Coarseness, SAE (Small Area Emphasis), and RMS (Root Mean Squared). Notably, the selected features include a mixture among geometric (Sphericity), neighboring gray tone difference (Coarseness), and texture features (both on small and large areas) comprehensively covering the different radiomic characteristics of the lesion. Both reduced models perform better than the full models because the excluded features degrade the classification (Fig. 5).

### Texture Models: Towards a Virtual Biopsy

Currently, biopsy is the standard technique for lesion classification and characterization, focusing on local and visual features of the sampled tissue. Hence, to simulate the biopsy, it would be sufficient to consider only the texture features of the segmented lesion. To investigate and compare the informative content of the texture and geometric features (e.g., Volume, Sphericity), the **RDF-ABVS Texture Model** and **RDF-DBT Texture Model** were introduced. These are the most effective models (i.e., RDF) that have been retrained to use only texture features to make their predictions. The Texture Models, even neglecting the significant contribution of geometric features, prove to train adequate classifiers (Fig. 5). Indeed, the AUC-ROC of the RDF-DBT Texture Model is 74.1% (compared to 89.9% of the RDF-DBT Model). Similarly, the AUC-ROC of the RDF-ABVS Texture Model is 71.8% (compared to 72.3% of the RDF-ABVS Model). Note that the ABVS/DBT performance gap is reduced when the geometric features are neglected.

## Discussion

In this work, we compared ABVS/DBT capability to classify benign/malignant breast tumors in a population of 66 women (69 lesions) using radiomic features. Three Machine Learning method were employed: Random Decision Forests, Support Vector Machines and Logistic Regression. They were trained and validated on a novel dataset of paired ABVS/DBT acquisitions using an ad hoc nested LOO cross-validation procedure. This approach allows us to avoid data leakage among training and validation sets and, consequently, to obtain a low-biased estimate of generalization capability of the model even with a small sample size. Furthermore, an adaptive selection strategy was successfully applied to obtain a minimal highly informative subset of features, so that derived models were computationally lighter and less affected by overfitting. The first major finding of this study is to highlight the greater effectiveness of ensemble methodology (RDF) to provide efficient prediction of tumor classification using radiomic features compared to single-prediction methods (Logit, SVM). RDF radiomic-based models for both ABVS and DTB acquisition prove to efficiently discriminate malignant/benign lesions (AUC-ROC: RDF-ABVS 72.3%, RDF-DBT 89.9%, using respectively 22/19 features). Nevertheless, even this reduced set of features is likely to contain redundant information. In fact, the adaptive selection strategy leads to a minimal subset of features with even greater classification power compared to the full set (AUC-ROC: ABVS 85.8% with 3 features, DBT 92.1% with 7 features). The latter suggests the importance of complementing classical radiomic analyses (based on hundreds or even thousands of features) with appropriate selection strategies to reduce the presence of confounding variables, especially in small/medium size datasets. As detailed in the “Results” section, independently of the set (or subset) of features used to train the classification model, using DBT data resulted in higher classification performances with respect to ABVS data, almost surely due to the image resolution. However, some kind of complementarity cannot be excluded: when comparing the predictions at a patient level, only 8.7% of lesions are misclassified by both the Full RDF Models. Finally, the removal of the (highly influential) geometric information from the model results in less accurate but still valid predictions (AUC-ROC: RDF-ABVS tx. 71.8%, RDF-DBT tx. 74.1%). This confirms radiomics as a tool capable of extracting information beyond the human eye. A limitation of this work is the size of the dataset (66 subjects, 69 lesions) deeply influenced by the difficulty of collecting reliable annotated images from DBT and ABVS covering the same lesions and by the time-consuming and demanding clinician-guided image segmentation process. Consequently, we focused on a binary classification task instead of a more complex tumor stage stratification. Such a limitation has been mitigated by LOO cross-validation and the use of the adaptive selection algorithm; of course, a larger study population will increase the statistical power of the results. In this respect, the ongoing activities of the P.I.N.K project will help, by collecting multimodal data from additional centers.

Future directions of research aim at the development of a mixed model of ABVS and/or DBT that includes also patient clinical history. In this perspective, a larger dataset is crucial also to tackle the more difficult task of a multi-class analysis, to enable both phenotype and tumor stage characterization. The results described and discussed above indicate that for BC the **virtual biopsy**, i.e., a radiomic-based ML, which uses only image data to characterize the lesion, is not so far.

## Data Availability

The PINK study data sharing is not applicable as specified in the informed consent signed by women.

## Footnotes

1 https://docs.scipy.org/doc/scipy/reference/ndimage.html

2 *Bin width* is the width of each bin in the histogram that represents the gray level intensities of the image.
